# Platelet-to-Albumin Ratio: The Prognostic Utility in the Prediction of 2-Month Postoperative Heart Transplant Complications

**DOI:** 10.3390/jcdd10060241

**Published:** 2023-05-31

**Authors:** Dragos-Florin Baba, Horatiu Suciu, Laurentiu Huma, Calin Avram, Alina Danilesco, Diana Andreea Moldovan, Andrei Stefan Opincar, Anca Ileana Sin

**Affiliations:** 1 Emergency Institute for Cardiovascular Diseases and Transplantation, 540142 Targu Mures, Romania; dragos-florin.baba@umfst.ro (D.-F.B.);; 2 Department of Cell and Molecular Biology, George Emil Palade University of Medicine, Pharmacy, Science and Technology of Târgu Mures, 540142 Targu Mures, Romania; 3 Department of Surgery, George Emil Palade University of Medicine, Pharmacy, Science and Technology of Târgu Mures, 540142 Targu Mures, Romania; 4 Department of Medical Informatics and Biostatistics, George Emil Palade University of Medicine, Pharmacy, Science and Technology of Târgu Mures, 540142 Targu Mures, Romania; 5 Faculty of Medicine, George Emil Palade University of Medicine, Pharmacy, Science and Technology of Târgu Mures, 540142 Targu Mures, Romania

**Keywords:** platelet-to-albumin ratio, monocyte-to-albumin ratio, heart transplant, complications, infections

## Abstract

Background: The platelet-to-albumin ratio (PAR), leucocyte-to-albumin ratio (LAR), neutrophil percentage-to-albumin ratio (NPAR), and monocyte-to-albumin ratio (MAR) represent easily reproducible markers, which may predict the outcomes in various diseases. Early postoperative complications might appear after heart transplantation, such as infections, diabetes mellitus type 2 (DM2), acute graft rejection, and atrial fibrillation (AFib). Objective: The aim of our study was to investigate the PAR, LAR, NPAR, and MAR values before and after heart transplantation, and the associations of the preoperative levels of these markers with the presence of postoperative complications in first two months after surgery. Methods: Our retrospective research was directed from May 2014 to January 2021, with a total number of 38 patients being included. We used cut-off values for the ratios from previously published studies, as well as our own determination of these levels by using a receiver operating characteristic (ROC) curve. Results: By ROC analysis, the optimal preoperative PAR cut-off value was 38.84 (AUC: 0.771, *p* = 0.0039), with 83.3% sensitivity, and 75.0% specificity. Applying a Chi square (χ^2^) test, PAR > 38.84 represented an independent risk factor for complications, regardless of cause, and postoperative infections. Conclusions: Preoperative PAR > 38.84 was a risk factor of developing complications of any cause, and postoperative infections in the first two months after heart transplantation.

## 1. Introduction

Cardiac transplantation remains a lifesaving therapy for patients with end-stage heart failure, despite novel therapies being improved in terms of medication and treatment strategies. On 3 December 1967, the world’s first human-to-human heart transplant was conducted by Christiaan Barnard in Cape Town, South Africa, on a 53-year-old male. In the first period following heart transplantation, the patient’s status was excellent. Later, a thoracic radiography revealed lung infiltrates, which were mistakenly interpreted as part of graft rejection. Intense immunosuppressive therapy was initiated, but unfortunately the patient died after 18 days as a result of a severe pneumonia [1].

Some of the most common reported postoperative complications are represented by graft rejection, which is reported to have the highest risk of development in the first six months following heart transplantation, as well as cardiac allograft vasculopathy (CAV), graft dysfunction, and infection, the latter having the highest incidence at 1 year [2,3].

Systemic inflammation has been recognized as a common pathological feature involved in heart diseases. Specifically, inflammation plays a central role in the development, progression and complications of chronic heart failure, being predictive of poor outcomes [4].

Essentially, based on the evidence that elevated levels of cytokines were observed in patients with heart failure, the evolution of this disease can be attributed to sustained pro-inflammatory cytokine signaling. As a result, pro-inflammatory cytokines can induce cardiomyocyte hypertrophy and apoptosis, leading to further extension of the inflammatory processes [5].

Several new hematological biomarkers, such as platelet count-to-albumin ratio (PAR), leucocyte count-to-albumin ratio (LAR), neutrophil percentage-to-albumin ratio (NPAR), and monocyte count-to-albumin ratio (MAR), that are easily reproducible, are increasingly approached in today’s research, with the potential of becoming predictive factors in the outcomes of various diseases. The clear advantage of using these biomarkers resides in the simplicity and ease of obtaining these ratios.

It has been already proven that a decrease in plasma albumin concentration associates an increased risk of cardiovascular diseases. This concept indicates that albumin has a fundamental and complex role in the development of pathological processes, such as atherosclerosis and thrombosis [6].

Serum albumin is a protein with antioxidant, immunomodulatory and detoxification functions, being responsible for 75% of the plasma oncotic pressure [7]. Synthesis of albumin can be reduced in a variety of situations, including poor nourishing, and inflammation. Hypoalbuminemia can be noticed in patients with inflammatory disorders, due to greater fractional catabolic rate, and increased vascular permeability [8,9].

The aim of our study was to investigate the values of PAR, LAR, NPAR, and MAR, before and after heart transplantation, and to analyze the association of the preoperative levels of these markers with the presence of postoperative 2-month complications. The previously studied cut-off levels for these markers, as well as our determined cut-off levels, were used in order to investigate the preoperative levels with the 2-month postoperative presence of complications regardless of cause, newly diagnosed type 2 diabetes mellitus (T2DM), paroxysmal documented atrial fibrillation (AFib), acute graft rejection, and infections.

## 2. Materials and Methods

### 2.1. Study Design and Patients

Our retrospective research was directed from May 2014 to January 2021, when a total number of 39 heart transplantations were performed in the Cardiovascular and Transplant Emergency Institute of Târgu Mureș. One patient was excluded from the group due to insufficient data evidence required for the purpose of this study. All participants have been handled and completed informed consent forms.

The study was conducted in accordance with the Helsinki Declaration and the ethics committee of Cardiovascular and Transplant Emergency Institute of Târgu Mureș, being the entity that approved the research protocol.

### 2.2. Management and Follow-Up

**Figure 1:**
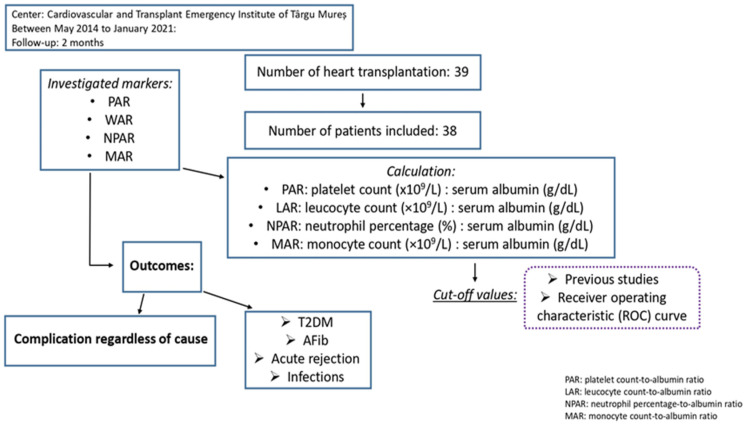
Study design.

For the patients included in the study, complete blood analysis before and after the transplantation, postoperative glucose level monitoring, periodic 12-lead electrocardiogram, right ventricle biopsies, and infection screening were obtained. The glucose values were determined by fasting glucose blood tests, and infection screening included samples from blood, urine and sputum in order to perform cultures. As part of our research, we analyzed the levels of PAR, LAR, NPAR, and MAR prior to and after the heart transplant, with a focus on the associations of preoperative biomarkers’ values with the incidence of complications in the first two months after the intervention. For the uniformity of the cut-off markers’ values, albumin concentration was evaluated as g/dL, and platelets as the blood count amplified by 10^9^/L. PAR was calculated as platelet count (×10^9^/L) divided by albumin serum concentrations (g/dL), LAR as leucocyte count (×10^9^/L) divided by albumin serum concentrations (g/dL), NPAR as neutrophil percentage divided by albumin serum concentrations (g/dL), and MAR as monocyte count (×10^9^/L) divided by albumin serum concentrations (g/dL) (Figure 1).

### 2.3. Statistical Analysis

Data processing was performed using MedCalc version 19 (MedCalc Software Ltd., Ostend, Belgium); thus, for quantitative data, mean values, standard deviation, maximum, and minimum values were determined. Normality testing was performed using the Shapiro–Wilks test [10] between values before and after heart transplantation, using a *t* test for parametric data and the Wilcoxon test for non-parametric data. Associations between previous cut-off levels of the markers and the presence of complications regardless of cause, T2DM, AFib, acute graft rejection, and postoperative infections, were analyzed by using logistic regression. A receiver operating characteristic (ROC) curve was determined to establish the values of PAR, LAR, NPAR, and MAR in predicting the presence of complications regardless of cause, and postoperative infections. The discrimination of the markers was assessed by the area under the ROC curve (AUC). The optimal cut-off points were rated by Youden’s index. Preoperative elevated biomarker values were correlated with the incidence of complications, of any cause, and infections by applying Spearman’s correlation coefficient. Independent associations were made with a Chi square (χ^2^) test. For all the tests, the significance threshold was set to 0.05.

### 2.4. Previous Researches That Examined LAR, NPAR MAR, and PAR Prognostic Roles

We included in our study a number of the 10 previous published studies that investigated the role of these 4 markers, in order to evaluate the same cut-off levels to the presence of complications of any cause or specific type of complication in heart transplants (Table 1).

PAR has already been proved to have an influence on the prognosis of multiple diseases, such as hepatocellular carcinoma, IgA nephropathy, peritoneal dialysis patients, and esophageal squamous cell carcinoma [11,12,13,14]. Another highly investigated marker is NPAR. The NPAR cut-off levels included in our study have been investigated in the prognosis of oral cavity cancer, bladder cancer, and cardiogenic shock [15,16,17,18,19] (Table 1). All the cut-off levels will be later analyzed in logistic regression.

## 3. Results

Our cohort consisted of 4 females (10.5%) and 34 males (89.5%), with a mean age of 41.21 years (SD = 13.71). The majority of the total subjects were type A (42.1%) or O (34.2%), with a mean body mass index (BMI) of 23.81 (SD = 5.18). The main diagnosis as indication for heart transplant has been the non-ischemic cardiomyopathy (47.4%), followed by the ischemic cardiomyopathy (21.1%). The other indications that we found have been the congenital (15.8%), valvular (7.9%), restrictive (5.3%), and hypertrophic (2.6%) cardiomyopathies (Table 2). Mean left ventricle ejection fraction before heart transplant was 26.54% (SD = 13.23), with a mean pulmonary pressure of 52.08 mmHg (SD = 15.70), and mean dimension of the left ventricle of 69.46 mm (SD = 13.80).

Regarding to leucocyte, neutrophil, monocyte, and platelet counts, the general mean counts were 9.00 (SD = 4.26), 6.51 (SD = 4.17), 0.77 (SD = 0.34), and 202.11 (SD = 62.61), respectively. Based on the fact that albumin concentration decreases with the increase in age, we divided our cohort at a cut-off of 40 years. The mean albumin serum concentration was 4.08 (SD = 0.56), being lower in patients under the age of 40 years, having mean values of 4.01 g/dL (SD = 0.37) versus 4.12 g/dL (SD = 0.46). When looking at the proportion of complications, 78.9% of patients presented complications of any cause (30/38). As specific type of complications, 21.1% of patients developed T2DM (8/38), 15.8% had documented episodes of paroxysmal AFib (6/38), 18.4% acute graft rejection (7/38), and 50.0% presented postoperative infections (19/38) (Table 2). The main pathogen involved in the postoperative infections, in 47.3% of the infection cases, was Staphylococcus aureus (9/19). The other pathogens detected were Candida species, Pseudomonas aeruginosa, Staphylococcus epidermidis, Staphylococcus haemolyticus, and Klebsiella pneumoniae. Our study involved other frequently reported complications, such as early postoperative acute kidney injury, need of hemodiafiltration, and moderate to large pericardial effusion (>10 mm). The minimum hospital stay for the patients included was 2 months, with an overall mortality rate at that point of 7.9%.

From the point of view of the immunosuppressive therapy, all patients had induction treatment with R-ATG (rabbit anti-thymocyte globulin) in combination with methylprednisolone, followed by maintenance therapy in triple combination with Tacrolimus (92.1%), mycophenolate-mofetil (92.1%) and prednisone (89.5%). As an approach to the prophylaxis of opportunistic infections at discharge, antibiotic treatment with Cotrimoxazole and Valganciclovir was administered.

Elevated postoperative values were seen in LAR, NPAR, and MAR, with a mean level of pre-LAR of 2.26 (SD = 1.15) versus post-LAR of 4.92 (2.25), pre-NPAR of 17.72 (SD = 4.19) versus post-NPAR of 27.19 (SD = 6.12), and pre-MAR of 0.20 (SD = 0.10) versus post-MAR of 0.28 (SD = 0.19). For NPAR, a parametric test for paired samples was carried out with a Student’s *t* test, and for the other 4 markers, a nonparametric test using the Wilcoxon test. The values of LAR, NPAR, and MAR were significantly increased postoperatively, with a significant decrease in the albumin serum concentration, and PAR (Table 3).

There was no statistically significant relationship between the previous studied cut-off levels of PAR, LAR, NPAR, and MAR and the presence of complications of any cause. When looking at specific types of complications, we observed that MAR > 0.14 was a risk factor of developing 2-month postoperative infections (OR: 9.68, 95% CI:1.01–91.95; *p* = 0.0480) (Table 4).

When looking at the cut-off levels of MAR, the distribution of patients revealed 28 patients (73.7%) in the group with MAR > 0.14, and 10 patients (26.3%) in the MAR < 0.14 group.

**Figure 2:**
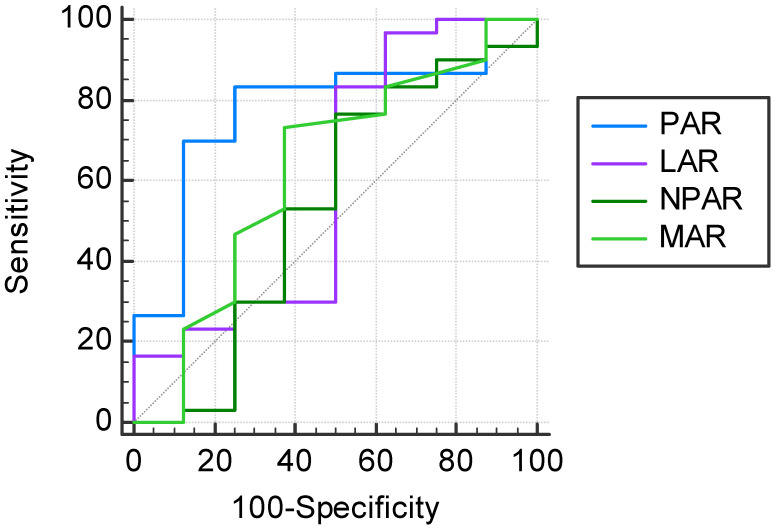
Prognostic values of PAR, LAR, NPAR, and MAR in the prediction of a 2-month complication of any cause after heart transplant.

The ROC analysis and Youden’s J-point suggested that the optimal cut-off values of PAR, LAR, NPAR, and MAR were 38.84, 3.17, 15.35, and 0.14, respectively, for the prediction of the 2-month apparition of postoperative complications regardless of cause after heart transplant (Figure 2).

When looking at the complications regardless of cause, PAR > 38.84 presented the highest AUC (0.771, 95%CI: 0.606–0.891), being statistically significant (*p* = 0.0039). The sensitivity of PAR > 38.84 was 83.3%, with a specificity of 75.0% (Table 5). In the distribution of subjects, considering PAR at the cut-off level of 38.84, we counted 27 patients above the cut-off (71.1%).

By performing χ^2^ test, we found independent statistically significant associations between PAR > 38.84 and the occurrence of complications regardless of cause (*p* = 0.0014). Furthermore, PAR > 38.84 was independently associated with the appearance of 2-month postoperative infections (*p* = 0.0135).

**Figure 3:**
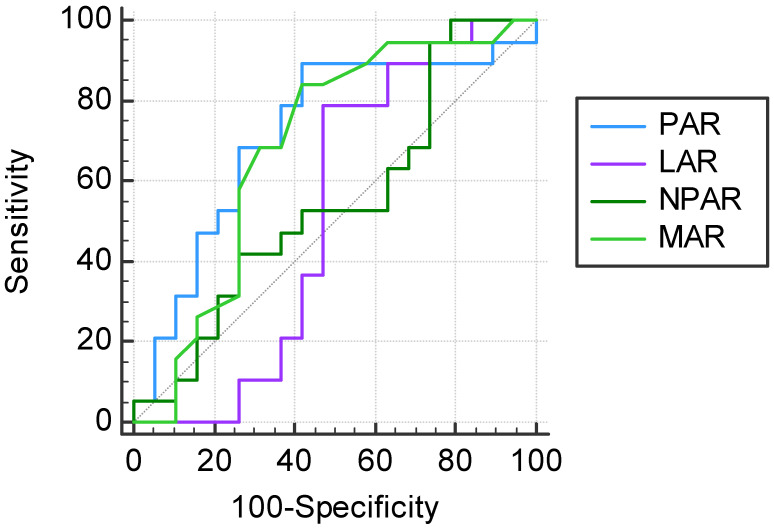
Prognostic values of PAR, LAR, NPAR, and MAR in the prediction of a 2-month postoperative infection after heart transplant.

The ROC analysis and Youden’s J-point suggested that the optimal cut-off values of PAR, LAR, NPAR, and MAR would be 40.26, 1.83, 20.17, and 0.15, respectively, for the prediction of the 2-month apparition of postoperative infections after heart transplant (Figure 3).

Regarding to our cut-off levels in the anticipation of postoperative infections, PAR > 40.26, and MAR > 0.15 presented AUC values of 0.720 (95% CI: 0.551–0.853, *p* = 0.0123) for PAR > 40.26, and 0.687 (95% CI: 0.516–0.827, *p* = 0.0429) for MAR > 0.15, respectively. We observed low specificity between these 2 cut-off values (57.9%), but with high sensitivity (89.5% for PAR, and 84.2% for MAR) (Table 6). The previous investigated cut-off level for MAR (MAR > 0.14) had a sensitivity of 84.2%, with a specificity of 52.6%. In logistic regression, PAR > 40.26 was significantly associated with postoperative infections, being a risk factor (OR: 21.68, 95% CI: 1.55–301.77; *p* = 0.0220). The same number of patients were in PAR > 40.26 group as in MAR > 0.15 group (25/38; 65.8%).

There were positive correlations between PAR, and MAR and the 2-month presence of postoperative complications of any cause, and infections. Even if these correlations were low-positive correlations, we found statistical significance between both markers and the presence of postoperative complications of any cause, and infections. High preoperative PAR values were significantly correlated with the presence of complications of any cause (*p* = 0.0177). No statistically significant correlation was found between LAR or NPAR and the presence of complications, regardless of cause, and infections (Table 7).

## 4. Discussion

One of the most intense focal points of medical research nowadays is represented by the postoperative outcomes of different factors, with a great impact on current therapeutic algorithms. One research area of great interest is posed by the preoperative inflammatory status. Thus, discovering new predictive indicators of positive or negative outcomes is of utmost importance for the improvement of current diagnosis and management guidelines. Some of the most important characteristics of a predictive tool is the high availability, repeatability and high sensitivity of the method, ensuring that, after validation, its advantages would benefit the maximum number of patients. Various studies aimed to find low-cost, repeatable investigations that could represent the foundation for a new predictive tool that could influence the management of multiple diseases. Routinely, platelet, leucocyte, neutrophil, and monocyte counts, in addition to albumin concentration levels, are determined preoperatively in patients who are to undergo heart transplantation. Ratios of these markers, e.g., PAR, LAR, NPAR, and MAR have been underlined as prognostic factors of several disorders.

In our study, the levels of LAR, NPAR, and MAR were significantly increased post-operatively, with a significant decrease in PAR. The initial cut-off values were imported from studies that have proven these parameters as a valuable indicator in the outcome of diseases. Based on the research of Zhang ZL et al. [20], who established a cut-off level of MAR of above 0.14, we found the highest risk of developing postoperative infections after heart transplantation in the first two months.

By ROC analysis and maximum Youden’s index, in the prediction of the complications regardless of cause, the optimal cut-off point for PAR was determined at the value of 38.84, with a statistically significant AUC, characterized by a sensitivity of 83.3%, and 75.0% specificity. PAR > 38.84 represented an independent risk factor for complications regardless of cause, in addition to being a risk factor for postoperative infections. Applying Spearman’s correlation coefficient, we observed a significant correlation between PAR levels and the presence of postoperative infections. In the ROC analysis, PAR > 40.26 showed higher AUC compared to the optimal cut-off value of MAR, with the same specificity (57.9%), but with higher sensitivity (89.5% versus 84.2%). By the use of logistic regression, with statistical significance, PAR > 40.26 presented the highest risk of postoperative infections compared with the other markers’ cut-off values. Staphylococcus aureus was identified as the main pathogen involved in associated infections. There were 3 deaths reported 2 months after heart transplantation, with a mortality rate of 7.9%, 2 of which were confirmed as a consequence of bacterial infection.

Hypoalbuminemia represents a risk factor in various cardiovascular diseases. Low serum albumin levels are associated with ischemic heart disease, heart failure, AFib, stroke, and venous thromboembolism [21]. In the case of heart transplant, it has been proven that hypoalbuminemia represents a poor prognostic factor, being associated with decreased survival rates [22,23]. In addition, as stated by a number of studies, hypoalbuminemia prior to kidney transplantation was associated with an increased risk of opportunistic BK, polyomavirus and cytomegalovirus infections [24].

In addition to their hemostatic role, platelets possess an important role in the inflammatory process. Multiple studies have shown that the progression of atherosclerosis can be attributed to the response of the activated platelets. It was demonstrated that platelets keep their activated status several years after heart transplantation [25]. In myocardial infarction, platelets are involved in the progression of coronary atherosclerotic plaques and the thrombotic occlusion of coronary vessels [26]. Cardiac allograft vasculopathy represents the long-term leading cause of morbidity, allograft failure and mortality in heart transplant patients. CAV is not fully understood, but immunological processes may play an important role in this post-transplant complication [27].

The ratio between the platelet count and albumin concentration has been proven to anticipate cardiac surgery-associated acute kidney injury, being associated with a worse prognosis in critical care patients [28].

The neutrophils involvement in cardiovascular diseases and complications has been intensively studied in the past years. Neutrophils are described as first-line defenders of the innate immune system, having an important role in mediating inflammation through a large phenotypic and functional heterogeneity. Neutrophils have developed a number of cellular adaptations to fulfill this role, such as phagocytosis, degranulation, apoptosis, the formation of neutrophil extracellular traps, and release of reactive oxygen species. Another key characteristic of this white blood cell subgroup is the capacity to respond to multiple signals by producing inflammatory factors [29,30].

Neutrophils are short-lived, highly mobile cells of the innate immune system that sense tissue injury in a matter of moments, being the first cells that take part in the defense process of the organism. As a result, they are responsible for the acute phase of the immune response, through the means of vascular adhesion and early arrival at the site of damaged tissue [31]. When there is a pathological response, neutrophils can promote inflammation, thus their increased activity is involved in the progression of cardiovascular diseases. Cytoplasmatic granules are released in the inflammatory processes, including enzymes such as myeloperoxidases, which initiate and maintain the course of fibrosis. Increased involvement of this enzyme is seen in the atrial tissue of AFib patients, and its increased serum levels are associated with AFib predisposition [32]. Previous studies involving NPAR showcased the importance of this parameter in several cardiovascular diseases and complications, such as ST-segment elevation myocardial infarction, free-wall rupture after an acute myocardial infarction, and its correlation with all-cause mortality in critically ill patients with coronary artery disease [33,34,35].

Regarding monocyte levels, it is important to note that these particular white blood cells play a crucial role in the innate immunity, being capable of differentiating into macrophages and dendritic cells [36]. The recruitment of monocytes has a key role in the organism’s defense against bacterial, protozoal, fungal and viral infections. Possible harmful effects during certain infections have been described in a number of studies [37]. Particularly, in severe acute respiratory syndrome coronavirus 2 (SARS-CoV-2) infection, the pulmonary macrophages derived from inflammatory monocytes become hyperactivated, ex-acerbating tissue damage at the site of infection [38]. There are three identified subpopulations of monocytes: classical monocytes, intermediate monocytes, and non-classical monocytes [39]. In a prospective study that included 104 patients who underwent cardiac surgery, elevated circulating intermediate monocyte levels were independently associated with extracardiac complications in the first 4 days after surgical intervention [40].

### Study Limitations

From a technical perspective, our study encountered a number of limitations. Firstly, specific limitations to the retrospective character of the research have to be mentioned, such as missing information, selection bias, and lost follow-ups [41]. Our second limitation is represented by the decreased number of patients in the cohort. Because of our country`s local legislation, the necessity of informed consent before organ transplantation, and lack of information on the topic, Romania has a lower record of heart transplantations [42]. The decreased number of subjects in the cohort may influence our results due to false-positive scores, with the possibility of overestimating the extent of the associations [43]. Furthermore, because of the decreased number of patients included, the AUC value may have larger discrepancies among the results [44]. Regarding complication documentation, unregistered, asymptomatic episodes of paroxysmal AFib are another important point of interest. Larger patient cohorts and further prospective studies are needed to increase the relevance and accuracy of our findings.

## 5. Conclusions

In our study, LAR, NPAR, and MAR were significantly increased postoperatively, with the albumin concentration and PAR being significantly decreased. Using the cut-off levels previously studied, we noticed that preoperative MAR > 0.14 was a risk factor of developing infections in the first 2 months after heart transplantation. By using our own cut-off levels of these markers, we observed that PAR > 38.84 represented an independent risk factor for the apparition of complications regardless of cause and postoperative infections.

Considering the complexity of this therapy option, and the need to optimize postoperative results, novel markers for predicting the risk of complications are essential. Further studies are needed in order to draw radical conclusions.

## Figures and Tables

**Table 1:** Cut-off values of PAR, LAR, NPAR, and MAR previously studied.

Cut-off Value	Author	Year of Publication	Title
PAR > 48	Li C, et al. [11]	2019	The preoperative platelet-to-albumin ratio predicts the prognosis of hepatocellular carcinoma patients without portal hypertension after liver resection
PAR > 60.8	Tan J, et al. [12]	2022	Platelet-to-albumin ratio: a novel IgA nephropathy prognosis predictor
PAR > 62.7	Yang Y, et al. [13]	2021	Platelet-to-albumin ratio: a risk factor associated with technique failure and mortality in peritoneal dialysis patients
PAR > 57	Huang Z, et al. [14]	2022	Prognostic significance of platelet-to-albumin ratio in patients with esophageal squamous cell carcinoma receiving definitive radiotherapy
LAR > 2	Lessomo FYN, et al. [15]	2023	The relationship between leukocyte-to-albumin ratio and atrial fibrillation severity
NPAR > 16.93	Ko CA, et al. [16]	2022	Prognostic value of neutrophil percentage-to-albumin ratio in patients with oral cavity cancer
NPAR > 18	Ferro M, et al. [17]	2021	Neutrophil percentage-to-albumin ratio predicts mortality in bladder cancer patients treated with neoadjuvant chemotherapy followed by radical cystectomy
NPAR > 23.54	Peng Y, et al. [18]	2020	Association between neutrophil-to-albumin ratio and mortality in patients with cardiogenic shock: a retrospective cohort study
NPAR > 25.3	Yu Y, et al. [19]	2020	The neutrophil percentage-to-albumin ratio as a new predictor of all-cause mortality in patients with cardiogenic shock
MAR > 0.14	Zhang ZL, et al. [20]	2021	Monocyte-to-albumin ratio as a novel predictor of long-term adverse outcomes in patients after percutaneous coronary intervention

**Table 2:** Baseline Characteristics of Heart Transplantation Recipients.

	Total (*n* = 38)	Age < 40 yrs (*n* = 13)	Age ≥ 40 yrs (*n* = 25)
BMI (kg/m^2^)			
• Mean (SD)	23.81 (5.18)	21.01 (5.75)	25.27 (4.29)
• Min	13.20	13.20	15.20
• Max	33.90	30.40	33.90
ABO blood type			
• O	13 (34.2%)	4 (10.5%)	9 (23.7%)
• A	16 (42.1%)	4 (10.5%)	12 (31.6%)
• B	4 (10.5%)	1 (2.6%)	3 (7.9%)
• AB	5 (13.2%)	4 (10.5%)	1 (2.6%)
Diagnosis			
• Non-ischemic (%)	18 (47.4%)	6 (15.8%)	12 (31.6%)
• Ischemic (%)	8 (21.1%)	1 (2.6%)	7 (18.4%)
• Congenital (%)	6 (15.8%)	3 (7.9%)	3 (7.9%)
• Valvular (%)	3 (7.9%)	1 (2.6%)	2 (5.3%)
• Hypertrophic (%)	1 (2.6%)	0 (0.0%)	1 (2.6%)
• Restrictive (%)	2 (5.3%)	2 (5.3%)	0 (0.0%)
Leucocyte count (×10^9^/L)			
• Mean (SD)	9.00 (4.26)	9.29 (5.06)	8.85 (3.89)
• Min	3.47	3.47	3.91
• Max	24.74	24.55	24.74
Neutrophil count (×10^9^/L)			
• Mean (SD)	6.51 (4.17)	6.46 (5.10)	6.54 (3.71)
• Min	2.09	2.09	2.94
• Max	22.40	22.27	22.40
Monocyte count (×10^9^/L)			
• Mean (SD)	0.77 (0.34)	0.95 (0.41)	0.67 (0.25)
• Min	0.17	0.41	0.17
• Max	1.89	1.89	1.19
Platelet count (×10^9^/L)			
• Mean (SD)	202.11 (62.61)	201.69 (67.46)	202.32 (61.38)
• Min	82.00	93.00	82.00
• Max	327.00	327.00	327.00
Albumin (g/dL)			
• Mean (SD)	4.08 (0.56)	4.01 (0.37)	4.12 (0.64)
• Min	2.20	3.50	2.20
• Max	5.00	4.60	5.00
Postoperative complications			
• Any cause	30 (78.9%)	11 (28.9%)	19 (50.0%)
• Type 2 DM	8 (21.1%)	2 (5.3%)	6 (15.8%)
• Paroxysmal AFib	6 (15.8%)	1 (2.6%)	5 (13.2%)
• Acute rejection	7 (18.4%)	1 (2.6%)	6 (15.8%)
• Infections	19 (50.0%)	8 (21.1%)	11 (28.9%)

**Table 3:** Preoperative versus postoperative values of Albumin, PAR, LAR, NPAR, and MAR.

	Pre-	Post-	*p*-Values
ALBUMIN			
• Mean (SD)	4.08 (0.56)	3.34 (0.63)	
• Min	2.20	1.70	<0.0001 *
• Max	5.00	4.80	
PAR			
• Mean (SD)	50.48 (17.97)	40.15 (14.91)	
• Min	20.75	18.33	0.0010 *
• Max	99.33	78.24	
LAR			
• Mean (SD)	2.26 (1.15)	4.92 (2.25)	
• Min	0.85	0.76	<0.0001 *
• Max	7.01	10.98	
NPAR			
• Mean (SD)	17.72 (4.19)	27.19 (6.17)	
• Min	8.73	10.91	<0.0001 *
• Max	27.88	48.35	
MAR			
• Mean (SD)	0.20 (0.10)	0.28 (0.19)	
• Min	0.03	0.05	0.0465 **
• Max	0.54	0.96	

* *t* test; ** Wilcoxon test.

**Table 4:** Association between preoperative PAR, LAR, NPAR, and MAR and the presence of complications using the cut-off values collected from previous studies.

	Complications OR/95% CI	Type 2 DM OR/95% CI	AFib OR/95% CI	Acute Rejection OR/95% CI	Infections OR/95% CI
PAR > 48	6.40 0.50–80.86	0.68 0.11–4.07	2.07 0.24–17.50	0.31 0.03–2.47	3.49 0.57–21.24
PAR > 57	1.42 0.09–21.44	0.88 0.11–6.75	0.91 0.11–7.44	0.16 0.01–1.86	5.23 0.74–36.98
PAR > 60.8	1.75 0.12–25.75	0.42 0.03–4.47	0.51 0.04–5.87	0.32 0.02–3.64	2.75 0.38–19.94
PAR > 62.7	- * -	0.40 0.03–4.35	0.58 0.04–7.25	0.35 0.03–4.18	2.64 0.36–19.30
LAR > 0.2	0.74 0.09–5.55	1.42 0.23–8.67	4.25 0.42–42.21	0.80 0.12–5.23	2.06 0.36–11.63
NPAR > 16.93	3.23 0.38–27.40	0.66 0.08–5.01	9.68 0.73–128.36	0.09 0.006–1.37	0.67 0.11–4.01
NPAR > 18	0.94 0.11–7.79	0.18 0.01–2.37	3.70 0.47–29.00	0.27 0.02–3.20	1.10 0.17–7.02
NPAR > 23.54	0.52 0.03–8.33	1.33 0.04–38.84	3.75 0.14–97.55	- * -	* -
NPAR > 25.3	0.83 0.04–16.99	* -	- * -	- * -	- * -
MAR > 0.14	0.43 0.05–3.74	7.63 0.45–127.48	0.23 0.02–2.61	- * -	9.68 1.01–91.95 *p* = 0.0480

* cannot be calculated.

**Table 5:** Area under the receiver operating characteristic (AUC ROC) curves of our 4 investigated markers (PAR, LAR, NPAR, and MAR) in predicting complications of any cause.

	Cut-Off	AUC	95% CI	*p*-Value	Sensitivity	Specificity
PAR	>38.84	0.771	0.606–0.891	0.0039	83.3	75.0
LAR	<3.17	0.600	0.429–0.755	0.4768	96.7	37.5
NPAR	>15.35	0.538	0.369–0.700	0.7913	76.7	50.0
MAR	>0.14	0.625	0.453–0.776	0.3341	73.3	62.5

**Table 6:** Area under the receiver operating characteristic (AUC ROC) curves of our 4 investigated markers (PAR, LAR, NPAR, and MAR) in predicting postoperative infections.

	Cut-Off	AUC	95%CI	*p*-Value	Sensitivity	Specificity
PAR	>40.26	0.720	0.551–0.853	0.0123	89.5	57.9
LAR	>1.83	0.518	0.350–0.683	0.8633	79.0	53.6
NPAR	<20.18	0.548	0.379–0.710	0.6178	94.7	26.3
MAR	>0.15	0.687	0.516–0.827	0.0429	84.2	57.9

**Table 7:** Correlations between preoperative PAR, LAR, NPAR, and MAR and the presence of postoperative complications regardless of cause, and infections.

	Complications			Infections		
	R-Value	95% CI	*p*-Value	R-Value	95% CI	*p*-Value
PAR	0.38	0.07–0.62	0.0177	0.38	0.07–0.62	0.0181
LAR	−0.14	−0.44–0.18	0.3975	0.03	−0.29–0.34	0.8525
NPAR	0.05	−0.27–0.36	0.7521	−0.08	−0.39–0.24	0.6161
MAR	0.17	−0.15–0.47	0.2881	0.32	0.005–0.58	0.0468

## Data Availability

The datasets generated and analyzed during the current study are available from the corresponding author on reasonable request.
